# Emerging Principles in the Transcriptional Control by YAP and TAZ

**DOI:** 10.3390/cancers13164242

**Published:** 2021-08-23

**Authors:** Alejandro Lopez-Hernandez, Silvia Sberna, Stefano Campaner

**Affiliations:** Center for Genomic Science of IIT, CGS@SEMM (Istituto Italiano di Tecnologia at European School of Molecular Medicine), Fondazione Istituto Italiano di Tecnologia (IIT), 20139 Milan, Italy; alejandro.lopez@iit.it (A.L.-H.); silvia.sberna@iit.it (S.S.)

**Keywords:** YAP, TAZ, WWTR1, hyper-transcription, epigenetic regulation, transcriptional addiction, transcriptional mechanism

## Abstract

**Simple Summary:**

YAP and TAZ are transcriptional cofactors that integrate several upstream signals to generate context-dependent transcriptional responses. This requires extensive integration with epigenetic regulators and other transcription factors. The molecular and genomic characterization of YAP and TAZ nuclear function has broad implications both in physiological and pathological settings.

**Abstract:**

Yes-associated protein (YAP) and TAZ are transcriptional cofactors that sit at the crossroad of several signaling pathways involved in cell growth and differentiation. As such, they play essential functions during embryonic development, regeneration, and, once deregulated, in cancer progression. In this review, we will revise the current literature and provide an overview of how YAP/TAZ control transcription. We will focus on data concerning the modulation of the basal transcriptional machinery, their ability to epigenetically remodel the enhancer–promoter landscape, and the mechanisms used to integrate transcriptional cues from multiple pathways. This reveals how YAP/TAZ activation in cancer cells leads to extensive transcriptional control that spans several hallmarks of cancer. The definition of the molecular mechanism of transcriptional control and the identification of the pathways regulated by YAP/TAZ may provide therapeutic opportunities for the effective treatment of YAP/TAZ-driven tumors.

## 1. Introduction

Yes-associated protein (YAP) and transcriptional coactivator with PDZ-binding motif (TAZ, also called WWTR1) are transcriptional coactivators, which were initially identified as downstream effectors of the Hippo signaling pathway [1,2]. The Hippo cascade is a highly conserved pathway that acts as a key regulator of organ size and tissue homeostasis and that, once dysregulated, contributes to tumorigenesis [3]. In higher eukaryotes, the core members of this signaling pathway are formed by the protein kinases Mst1/2 and Lats1/2, which, in collaboration with their cofactors Sav1 or Mob1, phosphorylate YAP/TAZ and repress their activity by preventing their nuclear translocation or by inducing their degradation in the cytoplasm [3,4]. Several other upstream regulators of YAP/TAZ have been identified [3,4,5,6], many of which are involved in cell polarity and cell adhesion signaling. During the establishment of the apical–basal polarity in epithelial cells, YAP/TAZ activity is modulated through the Hippo signaling core members by different protein complexes, including the apical crumbs complex (CRB) and the aPKC–PAR complex [7,8]. These are connected to the Hippo components through several proteins as the Angiomotin family (AMOTs) [9,10], neurofibromin 2 (NF2), and kidney and brain protein (KIBRA) [11,12]. Other polarity proteins, such as the Scribble complex (formed by Scribble, DLG, and LGL), act as a membrane-localized adaptors that sequester and inhibit YAP/TAZ [13,14]. The protocadherin Fat, which plays a key role in planar cell polarity, also activates the Hippo pathway [15,16]. Furthermore, several proteins related to cell adhesion and the formation of intercellular junctions, such as PTPN14, LIN7C, PATJ, MPDZ, E-cadherin, and α-catenin, have also been identified as repressors of YAP/TAZ [7,17]. Changes in the state and composition of the extracellular matrix (ECM), cytoskeletal tension, as well as cell shape, represent other pillars in the upstream control of YAP/TAZ activity. For instance, in a neoplastic context, changes in the microenvironment can induce aberrant mechanical signals that affect YAP/TAZ activity [18]. These mechanical cues are mostly transduced by the tension and organization of the F-actin cytoskeleton through the engagement of the Rho family of small GTPase [19,20,21,22]. YAP/TAZ are also modulated by other signaling pathways; for instance, the stimulation of G-protein-coupled receptors (GPCRs) by various ligands as lipids (lysophosphatidic acid and sphingosine-1-phosphophate) or hormones (glucagon or adrenaline) results in YAP/TAZ activation [23]. In addition, the WNT signaling, which is involved in cell proliferation, stem cell expansion, regeneration, and tumorigenesis, can also control YAP/TAZ activity. Indeed, YAP/TAZ are integral components of the destruction complex, an intracellular signaling hub that, by sequestering β-catenin and YAP/TAZ into the cytoplasm, targets these proteins for proteasomal degradation. Once Wnt ligands engage their cognate receptors, both YAP/TAZ and β-catenin are released from the destruction complex and shuttle to the nucleus to activate their respective target genes [24]. Of note, the WNT pathway can also regulate YAP/TAZ through mechanisms independent of canonical Wnt/β-catenin signaling [25,26]. Overall, YAP/TAZ are emerging as essential genes that are able to integrate chemical and mechanical signals in order to regulate tissue growth during development, regeneration, and cancer.

In this review, we will concentrate on the “nuclear” function of YAP/TAZ, with a particular focus on cancer. We will provide a detailed overview of the role of YAP/TAZ as transcriptional coactivators and their interaction with other transcriptional components. Then, we will summarize the main networks of transcription factors (TFs) that are controlled by and that interact with YAP/TAZ. Finally, we will present a panoramic view of the transcriptional programs regulated by YAP/TAZ in cancer. It should be pointed out that many of the cited studies focused on either YAP or TAZ and only some performed a comparative analysis on both cofactors. Thus, despite the broad functional redundancy of YAP and TAZ, specific findings may not necessarily concern both factors.

## 2. YAP/TAZ Are Transcriptional Cofactors

YAP/TAZ are defined as transcriptional cofactors, since they are endowed with transactivation activity but cannot bind DNA. Consequently, they need to form complexes with DNA-binding proteins in order to regulate gene expression. While several TFs have been reported to interact with YAP/YAZ (see paragraph 3), collective evidence indicates that the transcriptional enhanced associate domain (TEAD) TFs are the main partners which mediate both transactivation and growth-promoting activities [27,28,29,30,31,32]. In higher eukaryotes, there are four TEADs (TEAD1–4), which are highly similar in structure and function and show the partially overlapping of tissue-specific expression [33]. Their TEA/ATTS DNA-binding domain is located at the N-terminus and consists of a highly conserved 68 amino acid domain, which binds to the MCAT DNA motif (5′-CATTCCA/T-3′) [34]. Instead, their C-terminus harbors a protein–protein interaction domain that mediates the binding to YAP/TAZ, as well as to other binding proteins [27,28,29,30,31]. On the other hand, YAP/TAZ possess an N-terminally located TEAD interaction domain required for TEAD binding and a C-terminal transactivation domain [4]. TEADs are constitutively bound to chromatin and, in the absence of YAP/TAZ interaction, are associated with the VGLL1-4 co-repressors. Upon YAP/TAZ binding, VGLL1-4 are displaced, and thus, TEADs shift from being repressors to being either transcriptional activators or repressors [35]. A number of studies based on biochemical analysis of YAP/TAZ-associated factors, as well as genome-wide chromatin association analyses, have contributed to building a model of how the YAP/TAZ–TEAD complex controls transcription at target genes. The emerging picture indicates a prominent role for YAP/TAZ in promoting the enhancer-directed control of gene expression, coherently with their genomic distribution [36,37]. At enhancers, YAP/TAZ promote the recruitment of the mediator complex, which is required to establish enhancers–promoters contacts by long-range chromatin looping, possibly mediated by cohesins [37,38,39]. In addition, YAP/TAZ recruit the chromatin reader BRD4 and its paralogues [37]. BRD4 recruitment is mediated both by direct protein–protein interaction with YAP/TAZ and by the presence of acetylated chromatin. Following its recruitment, BRD4, which also has intrinsic histone acetyltransferase activity, acetylates K122 of the globular domain of H3, a chromatin mark that promotes RNA Pol II binding at promoters [40]. Whether the recruitment of RNA Pol II at promoters also depends on the mediator complex, which likely controls the assembly of the pre-initiation complex, is still an open question. Both the mediator complex and BRD4 trigger elongation by favoring the recruitment of the pTEFb complex, which, by phosphorylating the C-terminal domain of the initiating/stalled RNA Pol II on Ser2, triggers its release from the promoter, thus leading to productive elongation and transcription. Interestingly, the stability of nuclear YAP/TAZ is regulated by CDK7, which phosphorylates YAP/TAZ on sites Ser169/Ser128/Ser90. These phosphorylations prevent the interaction of YAP/TAZ with the CRL4-DCAF12 E3 ubiquitin ligase, therefore avoiding their Hippo-independent nuclear degradation [41]. CDK7 is a component of the TFIIH complex, a general TF, which promotes transcription initiation by phosphorylating the C-terminal domain of RNA Pol II on Ser5. This suggests a positive feedback regulation that reinforces YAP/TAZ-dependent transcriptional control by preventing their turnover at promoters. Thus, YAP/TAZ exert a multifactorial control of gene expression, which is based on the establishment of promoter/enhancer contacts and the recruitment and activation of RNA Pol II. Notably, this creates pharmacological dependencies in YAP/TAZ-driven tumors which open up the potential of targeting YAP/TAZ-dependent transcription at multiple levels. BRD4 inhibition, by preventing RNA Pol II loading, blunts the growth of YAP/TAZ-addicted breast tumors and YAP-driven liver tumors, and also rescues chemosensitivity in drug-resistant melanomas [37]. THZ1, a CDK7 inhibitor, suppresses the orthotopic growth of human triple-negative breast cancers and reduces liver growth and tumor burden in MST1,2 double-KO mice [41]. Flavopiridol, a CDK9 inhibitor, which suppresses RNA Pol II elongation, blunts YAP-driven liver overgrowth [39].

Less is known concerning YAP/YAZ-dependent gene repression. Most of the current knowledge comes from the study of Estarás and colleagues, which examined the transcriptional repression of mesendoderm genes during the early differentiation of human embryonic stem cells. By chromatin immunoprecipitation coupled to next generation sequencing (ChIP-seq) and GRO-seq, they observed that YAP–TEAD selectively counteract the Activin/SMAD2,3/Wnt-dependent induction of mesendoderm genes by inhibiting p-TEFb-dependent elongation and by recruiting the negative elongation factors NELF [42].

## 3. Interaction with Other TFs

**Figure 1:**
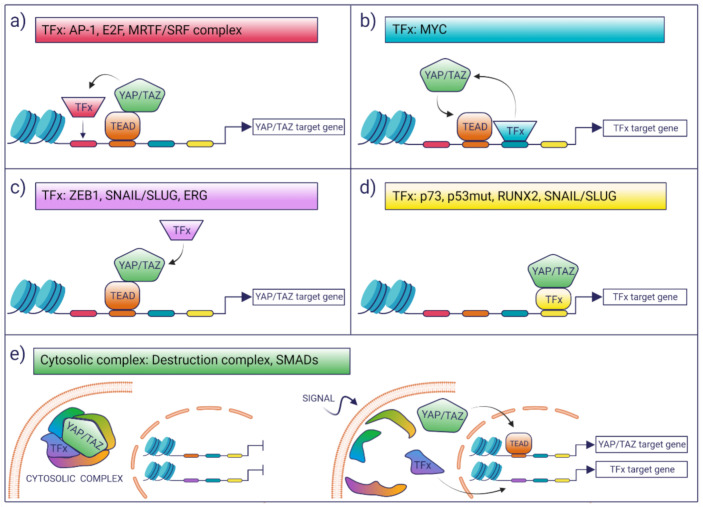
YAP/TAZ orchestrate transcriptional control by interacting with other transcription factors (TFs). A schematic overview of the general mechanisms by which YAP/TAZ can integrate with other transcription factors in order to modulate complex transcriptional responses. (**a**) Regulation of YAP/TAZ targets by *cis* interactions with other TFs; (**b**) regulation of other TFs targets by YAP/TAZ; (**c**) regulation of YAZ/TAZ activity on their targets mediated only by protein–protein chromatin-associated complexes; (**d**) modulation of other TFs activity, on their respective targets, mediated only by protein–protein chromatin-associated complexes; (**e**) modulation based on non-nuclear protein–protein interaction. Please note that although DNA regulatory elements have been placed near the transcribed genes, it is implied that these modes of regulation apply to both promoters and enhancers. Created with BioRender.com. (https://help.biorender.com/en/articles/3619405-how-do-i-cite-biorender, accessed on 23 July 2021).

Biochemical data and recent genome-wide chromatin studies indicate that YAP/TAZ interact with chromatin and regulate transcription primarily by associating with TEADs. Nonetheless, several reports document the association of YAP/TAZ with other TFs, suggesting the existence of intense transcriptional integration with other pathways for context-dependent transcriptional regulation. A review of the literature reveals emerging common themes in the way YAP/TAZ may integrate transcription (Figure 1):

(i) TFs may modulate the YAP/TAZ-dependent transcriptional landscape by boosting the activation of a subset of potential YAP/TAZ targets. This selection may be dictated in cis by the presence of cognate TF-binding motifs which favor the colocalization of YAP/TAZ with other TFs on proximal and distal regulatory regions, as is the case for AP-1 or MRTF/SRF [43,44].
(ii) TFs may enhance the binding of YAP/TAZ to TEAD loci that would otherwise be low-affinity sites for YAP/TAZ binding. This is the case of MYC target genes, whereby MYC promotes the recruitment of YAP/TAZ on constitutive TEAD-bound loci, which, in the absence of MYC expression, are not bound or regulated by YAP [45].
(iii) Co-operating TFs might directly interact with YAP/TAZ-TEAD and can be recruited to YAP/TAZ-bound loci in a manner that is independent of the presence of their cognate DNA motif, as is the case of ZEB1 [46]. In these latter cases, the open question is what are the factors that account for the selective interaction of these TFs with a subset of YAP/TAZ–TEAD-bound loci. A possibility is that these are mediated only by protein–protein interaction and that the topology or the composition of the chromatin-associated complexes dictates selective association and transcriptional regulation.
(iv) There are a number of examples where YAP/TAZ have been proposed to regulate transcription in a TEAD-independent manner, as is the case of p73, mutant p53, and the regulation of osteogenic programs by RUNX2 and SNAIL/SLUG [47,48,49,50]. In these instances, YAP/TAZ would function as transcriptional modulators of other transcriptional programs.
(v) The chromatin-independent association of YAP/TAZ can also contribute to the integration/coordination of transcriptional responses. This is the case of β-Catenin and YAP/TAZ, which are both components of the WNT destruction complex or the cytoplasmic association of YAP/TAZ with SMADs [7,51].

Thus, the interaction with other TFs can lead to the remodeling and potentiation of YAP/TAZ-dependent transcription, the co-regulation of common target genes shared with the other TFs, the concerted activation of distinct transcriptional programs, or the enhancement of the activity of other TFs on their target genes. The following is a summary of the most relevant interaction of YAP/TAZ with TFs and cofactors.

*AP-1*. Activator protein 1 (AP-1) is a group of dimeric TFs composed of JUN, FOS, and ATF family proteins. AP-1 proteins belong to the basic leucine zipper (bZIP) family and bind DNA as either homo- or heterodimers. Both gain- and loss-of-function studies have revealed specific roles for individual AP-1 components in cell proliferation, differentiation, apoptosis, and other biological processes. ChIP-seq analyses show that a high fraction of YAP/TAZ–TEAD peaks (70–80%) colocalizes with AP-1 and contains both a TEAD and an AP-1 motif [43,44]. Co-immunoprecipitation experiments indicate the physical interaction of TEAD with AP-1, thus suggesting that both the proximity of the cognate DNA-binding motifs and the protein–protein interaction of the two DNA-bound complexes contribute to gene regulation [43]. YAP/TAZ–TEAD–AP1 complexes are prevalent binding enhancers that regulate the expression of genes involved in cell cycle control and cell proliferation In addition, they regulate the activity of the DOCK-RAC/CDC42 module and drive the expression of genes controlling cell migration and invasion [44]. On these genes, the TEAD–YAP/TAZ–AP-1 complex recruits the NCOA coactivator, which is needed for the stabilization of the complex and transactivation [44]. In vivo genetics confirm reciprocal requirements of AP-1 and YAP/TAZ in tumorigenesis [43,52,53]. Considering that AP-1 activates signal-dependent enhancers by recruiting the SWI–SNF(BAF) chromatin remodeling complex, within the YAP/TAZ-TEAD-AP1, AP-1 could function as a pioneer factor that may allow the control of cell differentiation and lineage choice specification during development [54].

*E2F*. E2F TFs are the final effectors of the cyclin-dependent kinase (CDK)–RB–E2F axis, which drives cell cycle progression. Mutations in this pathway are frequent in cancer and lead to uncontrolled DNA replication and cell division. In Drosophila, the YAP/TAZ orthologue Yorkie (Yki) and dE2F1 share the binding of promoters of a large fraction of genes that are cooperatively activated during development. This concerted control of cell growth genes explains the pleiotropic effect of loss of function mutations of dE2F1 over Yki activation [55]. Co-regulation by YAP and E2F is confirmed in higher eukaryotes by studies on RAS-driven pancreatic cancer models: in a subset of tumors, RAS-induced oncogenic addiction is suppressed by the genetic selection of focal YAP amplification, which leads to the upregulation of E2F-dependent genes involved in cell cycle progression and DNA replication [56].

*MYC*. c-MYC is a basic Helix-Loop-Helix leucine-zipper TF that dimerizes with MAX to regulate gene expression. In physiological conditions, it is activated by growth factor stimulation, thereby controlling the expression of a large number of genes involved in cell growth and metabolism [57,58]. In cancer, MYC and its family members are frequently activated by genomic rearrangements or by upstream oncogenic signaling in order to amplify transcriptional responses of select pathways [57,58]. MYC-bound loci (promoters and enhancers) frequently colocalize with TEAD-bound regions [45]. MYC promotes the recruitment of YAP on these share loci, which in the absence of MYC expression are otherwise low-affinity binding sites for YAP. In turn, the recruitment of YAP boosts MYC-dependent transcription by promoting RNA Pol II pause-release. This co-regulation allows the integrated control of the expression of MYC targets in response to mechanical and biochemical cues and accounts for the cooperation of these two oncogenes in driving oncogenic transformation [45].

*Myb–MuvB complex*. The Myb–MuvB complex is a master regulator of genes expressed at the G2 and M phase of the cell cycle, which complements the activity of E2F factors in the control of cell cycle progression. During the S-phase, the MuvB complex (formed by five subunits LIN9, LIN37, LIN52, LIN54, and RBBP4) dissociates from the repressive DREAM complex (pRb, E2F4, and DP1) to bind to b-Myb. This Myb–MuvB complex then activates genes-regulating mitosis and cytokinesis. The expression of these genes is controlled by YAP/TEAD, which engage distal enhancers by binding b-Myb. Both complexes promote enhancer–promoter contacts required for transcriptional activation [59]. b-Myb is also a YAP/TAZ target gene, thus suggesting positive feedback regulation.

*MRTF/SRF*. Myocardin-related factors (MRTFs) are G-actin-binding coactivators that localize mainly in the cytosol in resting cells. As a result of mechanical (i.e., actin polymerization) or biochemical stimulation (i.e., growth factors), MRTFs shuttle to the nucleus where they bind to their cognate TF, the serum response factor (SRF). The MRTF/SRF complex drives the expression of genes relevant for fibrosis, including ECM proteins, integrins, cytokines, many components of the actomyosin cytoskeleton, through the binding of CC(A/T-rich)6GG cis-elements, called the CArG boxes. Genome-wide chromatin association studies in fibroblast reveal the co-regulation of genes by MRTF/SRF and YAP/TAZ-TEAD, which is mainly driven by the co-presence of their cognate DNA-binding motifs on promoters and enhancers. This creates codependency in the expression of these common target genes, whereby the expression and the activity of one of the two complexes are necessary and sufficient to activate the other TF complex, thus leading to gene transactivation [60]. This crosstalk depends on the ability of YAP/TAZ and MRTFs to activate reciprocal upstream regulators [60]. In addition, MRTFs are also shown to physically interact with YAP/YAZ, thus potentiating their metastatic activity in breast cancer cells [61]. Given their complementary function in gene regulation and analogies in their upstream regulation, MRTF/SRF, and YAP/TAZ-TEAD pathways may integrate upstream cues to coordinate cytoskeletal dynamics, fibrotic responses, and cell migration.

*RUNX*. The Runt-domain TFs (RUNXs) are generally involved in lineage differentiation and cancer development, although with functions that depend on the context and the specific RUNX involved. RUNX2, which controls mesenchymal stem cells differentiation along the osteogenic lineage, is upregulated during the epithelial-to-mesenchymal transition (EMT) and its expression correlates with poor prognosis in breast cancer [62]. RUNX2 interacts with both YAP and TAZ via their WW domain. The cooperative regulation of gene expression by YAP/TAZ and RUNX promotes osteoblastic differentiation [63], cell transformation, and stem cell self-renewal [64]. On the contrary, RUNX3, a tumor suppressor gene in gastric cancer, represses YAP/TAZ activity by forming a trimeric complex with YAP/TEAD, which prevents TEAD binding to DNA [47].

*TRPS1*. TRPS1 is a transcriptional repressor that belongs to the family of GATA TFs. These TFs are able to act as pioneers which regulate enhancer functions by altering their chromatin accessibility. In breast cancer cells, where TRPS1 is frequently overexpressed and amplified, TRPS1 physically interacts with TEAD and colocalizes with TEAD-bound loci. On these loci, TRPS1 promotes the recruitment of corepressor complexes (CTBP2, NCOR1, and NCOR2) along with their associated histone deacetylases (HDAC1 and HDAC3). This leads to the loss of H3K27ac at promoters and enhancers, loss of accessibility, and decreased promoter–enhancer contact, all of which result in the repression of YAP-dependent genes [65]. The overexpression of TRPS1 promotes aggressiveness in breast cancer models, possibly suggesting that pruning YAP-dependent transcription may provide growth and survival advantages to cancer cells.

*ZEB1*. ZEB1 is a transcriptional repressor known for its regulation of cellular motility, survival stemness, and EMT. In cancer, ZEB1 has been implicated in the regulation of early dissemination, metastasis, and therapy resistance. In triple-negative breast cancer, its association with YAP/TEAD enhances the transcription of a subset of YAP/TAZ targets. The binding of ZEB1 to the WW domain of YAP is required to target ZEB1 to these genes, while the presence of the E-box, which is the ZEB1 cognate DNA-binding motifs, is dispensable [46]. This is YAP specific, since no evidence of binding or co-regulation is found when TAZ is analyzed. ChIP-seq revealed that YAP/ZEB1 sites are also bound by AP-1, thus suggesting that ZEB1 co-regulates a subset of YAP/AP-1 targets [66].

*SNAIL and SLUG*. SNAIL and SLUG are TFs known to regulate EMT and the differentiation of the mesenchymal lineage and cancer stem cells. Their function is essential for skeletal stem/stromal cells’ self-renewal, differentiation, and proper bone development. These osteogenic programs are coordinated by the formation of protein complexes that activate the transcriptional activity of YAP/TAZ. Protein–protein interactions are mediated by the WW domain of YAP/TAZ and the repressor domain of SNAIL/SLUG [50]. These interactions potentiate both the YAP/TAZ transactivation of their canonical targets via TEAD and the expression of osteogenic genes via SLUG/SNAIL-TAZ interaction with RUNX2 [50]. Considering the co-expression and activity of YAP/TAZ and SNAIL/SLUG and RUNX2 in other contexts, this regulatory network might be generally conserved. It is also interesting to note that both ZEB1 and SLUG/SNAIL, which in their canonical function are repressors, in association with YAP/TAZ are converted into transcriptional activators [50].

*NICD/RBPJ*. Notch signaling is regulated by the interaction of membrane-bound ligands that engage Notch receptors on juxtaposed neighboring cells, leading to the proteolytic release of the Notch intracellular domain (NICD) fragment from the membrane. NICD translocates to the nucleus and binds the TF recombining binding protein suppressor of hairless (RBPJ) and the nuclear effector Mastermind-like, thus forming a complex that activates the transcription of the Notch target genes. YAP/TAZ and Notch crosstalk at different levels due to the YAP/TAZ-mediated transcription of Notch ligands and receptors and the YAP/TAZ–NICD co-regulation of common genes [67]. This is exemplified by their concerted regulation of CDX2, a lineage-specific TF required for trophectoderm differentiation. During early embryogenesis, Hippo signaling (through YAP/TEAD4) and Notch signaling (through NICD) control the expression of CDX2 in the blastocyst outer layer by regulating the activity of a cis enhancer [67]. Similarly, during embryonic development, YAP is recruited by the NICD/RBPJ in a TEAD-independent way to an enhancer that regulates the expression of Jagged1 (one of the Notch targets) [68]. This interaction is mediated by the first YAP WW domain. Given that other Notch targets are not affected, this co-regulation seems to be restricted to a subset of Notch target genes. These examples suggest that Hippo and Notch’s convergent regulation of gene expression programs involves the co-activation of distal regulatory elements.

*ERBB-4-ICD*. EGFR family member v-Erb-b2 avian erythroblastic leukemia viral oncogene homolog 4 (ERBB-4) is a receptor protein tyrosine kinase, which undergoes proteolytic processing by membrane proteases (γ-secretase) in response to ligands. The resultant soluble intracellular domain (ICD) of ERBB-4 is translocated to the nucleus, functioning as a transcriptional regulator [69]. The PPxY motif of ERBB4–ICD interacts with the WW domain of YAP, thus forming a ternary complex containing TEAD, which potentiates YAP/TEAD transactivation. This complex could mediate the oncogenic signaling of ERBB4–ICD and promote aggressiveness and migratory properties in cancer cells [66]. The ERBB4–ICD complex is inhibited both by Hippo signaling and by WWOX oxidoreductases, which compete with ERBB4–ICD for the binding to YAP, thus potentially accounting for WWOX tumor-suppressive activities in osteosarcomas [70].

*β-catenin*. β-catenin (βCAT) is a TF that is regulated by the WNT signaling pathway. Once stabilized, it shuttles to the nucleus, where it associates with LEF/TCF TFs to activate gene expression. It is required for a wide range of developmental processes, and it is frequently activated in cancer cells. The WNT/βCAT pathway and YAP/TAZ are intertwined at many different levels, both in the cytoplasm where their upstream regulation occurs and in the nucleus where they activate gene expression. In the developing heart, YAP and β-catenin co-regulate the expression of SOX2 and SNAI2, two key TFs that support cardiomyocytes proliferation by binding their respective cognate DNA motifs found at the SOX2 and SNAI2 loci [51]. Similar findings indicate that YAP/β-catenin regulate the expression of anti-apoptotic genes in cancer cells, along with TBX5 [71].

*TBX5*. TBX5 is a member of the T-box family of TFs (TBXs), characterized by a conserved 180 amino acid DNA-binding domain (T-box). TBXs regulate a variety of developmental processes, including specification of mesoderm, development of the heart, vasculature, and limbs, and tumorigenesis. Evidence suggests the transcriptional cooperation of YAP/TAZ and TBX5 in a β-catenin-independent way. In the developing heart, YAP/TAZ and TBX5 co-regulate the expression of cardiac genes by favoring the recruitment of histone acetyltransferases. Mutations of TBX5, which are identified in the Holt–Oram syndrome, impair TAZ binding and hamper gene activation [72]. This suggests a modality for the integrated transcriptional control of morphogenic genes expression programs during development.

*SMADs*. SMADs are TFs that are regulated by the superfamily of TGF-β and BMPs pathways. These signal transduction pathways regulate the cell cycle and differentiation and are altered in cancer. Activatory SMADs, SMAD2,3 (TGF-β-dependent), and SMAD1,5,8 (BMPs-dependent), once activated, associate with SMAD4 and translocate to the nucleus to activate transcription. Negative feedback regulation depends, in part, on their binding to the inhibitory SMAD6,7. Hippo signaling, YAP/YAZ activation, and TGF-β signaling intersect at different levels, in part by the association of YAP/TAZ with SMADs, which is regulated by the Hippo pathway and restrains SMADs within the cytoplasm [7]. Evidence for a direct control of gene expression comes from biochemical and genome-wide chromatin association studies in embryonic stem cells, where SMAD3, YAP/TAZ–TEAD, and OCT4 form a repressive complex (TSO), which recruits the NurD complex on genes associated with both pluripotency and differentiation towards the mesendoderm cell fate. Accordingly, the inactivation of the TSO complex prevents embryonic stem cell differentiation [73]. Given the genetic evidence supporting the requirement of YAP/TAZ, which by integrating mechanical signaling, supports BMP-directed osteogenic differentiation [74], it will be relevant to establish whether SMADs and YAP/TAZ may co-operate in cis to activate gene expression.

*ERG*. ERG belongs to the family of ETS TFs. Several observations support the oncogenic role of ERG and other ETS factors in prostate carcinogenesis [75]. ETS-family gene rearrangements occur in 20–50% of all human prostate adenocarcinomas. Modeling ERG activation in the prostate epithelium reveals that it could function as a cofactor for YAP by physically associating with YAP/TEAD to favor gene activation in part by increasing chromatin acetylation at H3K9/14. ERG-dependent YAP activation is required for cell transformation and invasion, and the overexpression of activated YAP phenocopies ERG activation. The evidence indicating that ERG could induce the transcription of YAP suggests a potential positive feedback control [76]. This illustrates how YAP/TAZ may represent general downstream targets of upstream oncogenic signals which will act directly on YAP/TAZ-dependent gene regulation.

*FOXO1*. Evidence linking YAP activity to the cellular responses to oxidative stress and its cytoprotective role during recovery from oxidative damage leads to the analysis of FOXO1. FOXO1 belongs to a family of TFs possessing a conserved DNA-binding domain termed the “Forkhead box”. ChIP on selected regulatory regions of the MnSOD, a critical gene in the antioxidant response, reveals that both YAP and FOXO1 bind and regulate MnSOD expression. In vivo genetic analysis shows that FOXO1 and YAP prevent cardiac cell death in response to oxidative stress [77]. FOXO1 was also recently identified as a downstream TAZ target in glioblastoma stem cells [78]. Future genome-wide studies may shed light on whether this will extend to all FOXO1 targets and whether this might be relevant in other settings as, for instance, cancer development.

*PITX2*. Paired-like homeodomain transcription factor 2 (Pitx2) is found to be upregulated in the border-zone ventricular cardiomyocyte nuclei after infarction in Hippo-deficient mice and is required for YAP-induced regenerative responses [79]. Genomic analysis reveals the enrichment for TEAD and PITX2-binding motifs on accessible genomic sites marked by H3K4me1 (presumably enhancers). In addition, ChIP-seq analysis reveals a subset of promoters of genes that are co-regulated by both YAP and PITX2. These genes are implicated in the anti-oxidant response and might account for the requirement of both YAP and PITX2 in heart regeneration [79].

*RELA (p65)*. RELA associates with either NF-kB1,2 to form the transcriptionally competent NF-kB complex. This complex is the effector of signals transduced following stimulation by a number of factors, including TNFa, TLRs, and TCR/BCR. The p65 subunit (RELA) of the NF-kB complex is shown to bind YAP/TEAD in response to TNFα stimulation. This leads to the assembly of the YAP/TEAD/p65 complex, which activates the expression of chemokines (IL-6 and CCL2) and glucose metabolism genes. This program supports migration in breast cancer cells, suggesting the crosstalk between NF-kB signaling and YAP/TAZ in cancer cells [80]. Previous data demonstrating TEAD/p65 association and the regulation of the MnSOD gene (through the activation of enhancers) may suggest broader transcriptional regulation [81].

*Mutant-TP53 (Gain of function)*. TP53 is a tetrameric TF that regulates cellular responses to stress conditions such as DNA damage, metabolic, and activation of oncogenes. It is frequently inactivated by loss of function mutations, which impair its tumor-suppressive activity. TP53 can also acquire the gain of function mutations which are positively selected during tumor evolution due to their pro-oncogenic activities. Among these activities, the mutant-TP53 gain of function promotes cell cycle by regulating transcription for a subset of cell cycle genes which are co-activated by NF-Y and YAP. On these genes, the presence of a CAAT motifs consensus sequence for NF-Y binding is required to recruit the mutant-TP53/NF-Y/YAP trimeric complex, which stimulates transcription by enhancing promoters acetylation [49].

*TP73*. TP73 belongs to the TP53 family and shares tumor-suppressive activity functions with TP53. Several reports show the association of p73 with YAP to form transcriptionally active complexes. YAP acts as a competitor of the ITCH E3 ubiquitin ligase, which binds and targets p73 for ubiquitin-mediated degradation [48]. The binding of YAP to the PPPY motif of p73 antagonizes ITCH and promotes p73 stabilization and its nuclear translocation. YAP/p73 complexes co-regulate p73 targets genes and exert tumor-suppressive activity in multiple myelomas via their c-Abl-dependent activation [82]. Their tumor suppressive function is mediated by the control of the expression of proapoptotic genes, such as BAX, PIG3, and p53AIP [83,84].

## 4. YAP/TAZ Regulate Enhancers

Initial studies concerning the activity of YAP/TAZ as transcriptional coactivators were focused on the analysis of the promoter-driven transcription of a few available target genes (e.g., CTFG and CYR61) [28,85]. This view was radically changed by the implementation of high-resolution and high-throughput technologies, as ChIP-seq, which allow the genome-wide mapping of YAP/TAZ-bound loci in several systems [36,39,43,86]. These studies provided unequivocal evidence that YAP/TAZ prevalently bind genomic regions that are distal to promoters and which are, by enlarge, marked by histone posttranslational modifications such as H3K27ac and H3K4me1. These are typical chromatin marks found at active enhancers [36,39,87,88]. The high overlap between YAP and TAZ ChIP-seq peaks determined in cells where the two factors are co-expressed suggests that they follow the same “rules of engagement” at genomic sites, consistently with their biochemical similarities and the shared DNA-binding partners [39,43]. In general, YAP/TAZ-bound loci show a strong enrichment of TEAD-bound DNA sequence motifs and largely overlap with TEADs’ ChIP-seq signals, thus indicating that TEADs are the main mediators of YAP/TAZ binding to DNA [28,36,43,45]. It is worth noting that the number of genomic loci bound by TEAD can exceed YAP/TAZ-bound loci by a factor of 10, thus implying that context may also shape the genomic distribution of YAP/TAZ and may dictate which TEAD-bound loci are bound by YAP/TAZ [45]. Notwithstanding that TEADs are preferential YAP/TAZ partners at enhancers, binding motif analysis at YAP/TAZ bound sites suggests that their association to chromatin could also be mediated by other TFs such as p73, mutant p53, and Snail/Slug [49,50,64]. While YAP/TAZ-bound enhancers are typically epigenetically bookmarked and accessible, there are instances where YAP/TAZ activity may require enhancer activation, as during cell fate-determination. Enhancer activation entails the eviction of nucleosomes to establish a region of ~300–400 bp of open chromatin that is permissive for TFs binding [89]. The mechanisms through which YAP/TAZ dynamically select these enhancers remain largely unknown. Current models emphasize the role of lineage-determining TFs with pioneering functions, which, by remodeling chromatin at poorly accessible regions, allow the recruitment of lineage specifying TFs [90]; thus, it is likely that YAP/TAZ will act in concert with such pioneering factors. Considering that AP-1 remodels nucleosome-occluded enhancers by recruiting the chromatin-remodeling SWI/SNF complex [54] and that AP-1 is a major partner of YAP/TAZ at enhancers [43], it is reasonable to assume that AP-1 may function as a pioneer factor for YAP/TAZ.

Some common themes are emerging concerning the type of enhancers regulated by YAP/YAZ in development and cancer. Consistently with their activity as de-differentiating factors, YAP/TAZ are able to bind and regulate developmental and lineage specification enhancers. For instance, during pancreatic development, YAP/TEAD regulate the activity of a subset of temporarily regulated enhancers. On these enhancers, YAP/TEAD integrate the combinatorial control of gene expression along with other lineage-specific TFs as HNF1B, ONECUT, PDX1, FOXA1, and GATA6, which colocalize with TEAD on co-regulated enhancers. This suggests a central role for YAP as a signal responsive regulator of multipotent pancreatic progenitors [91]. In addition, during embryonic development, the YAP–TEAD complex can bind tissue-specific enhancers of neural crest cells to temporally drive their delamination and migration by controlling the expression of EMT-related genes, such as SOX9, ZEB2, ETS1, SNAI2, and MYCN [88]. Another example concerns the dynamic regulation of lineage-specific enhancers involved in hepatocytes differentiation. Based on the occupancy of two key hepatic TFs, HNF4A and FOXA2, liver developmental enhancers can be classified as embryonic, exclusively adult, or continuously active. The ectopic expression of YAP in adult hepatocytes drives their de-differentiation into embryonic hepatoblasts and re-shuffles HNF4A and FOXA2 binding from adult enhancers to embryonic ones [92]. These data suggest that YAP/TAZ may orchestrate enhancer switching, thus dynamically tuning the differentiative status of somatic cells.

ATAC-seq analysis performed on adult cardiomyocytes reveals that, once they are reprogrammed by YAP into fetal-like progenitors, these cells lose nucleosomes and acquire open chromatin regions at a subset of YAP-bound loci. These genomic sites show a significant gain of topological contacts between promoters and enhancers, supporting the hypothesis that YAP overexpression alters local chromatin conformation by enhancing the looping between YAP-bound distal regions and promoters of target genes [93]. The newly accessible genomic loci are mainly developmental cardiac enhancers, which, once activated, drive the expression of cell cycles and embryonic genes, which progressively lead to heart hyperplasia [93]. A similar gain in chromatin accessibility driven by YAP/TAZ may also be relevant in cancer. For instance, the invasive phenotype of melanomas is associated with the broad chromatin remodeling of regions enriched for TEAD binding sites. TEAD silencing dramatically impairs invasive properties and cell viability of melanoma cell lines, thus suggesting a pivotal role for YAP/TAZ in the epigenetic control of the metastatic phenotype [94].

It is worth mentioning that there are also enhancers that are bound and repressed by YAP/TAZ. This aspect, which to date has received less attention, suggests context-dependent transcriptional regulation by YAP/YAZ. During the early stages of embryonic development, YAP/TAZ recruit the NuRD repressor on “switch-enhancer elements” bound by TEAD, SMAD2/3, and OCT4, thus preventing the expression of the mesendoderm specification genes [73]. This indicates that the YAP-dependent repression of these enhancers is a molecular switch that controls pluripotency and fate choice in ES cells.

In cancer cells, YAP/TAZ have been mapped to a subset of super-enhancers mainly associated with the expression of cell growth genes [37,39]. Super-enhancers are a subset of genomic loci heavily bookmarked by activatory histone marks, which supports high transcriptional rates of genes linked to oncogenic growth and cell identity [95,96,97]. The binding and regulation of a good fraction of super-enhancers in cancer cells may account for the transcriptional addiction imposed by YAP/TAZ [37,39]. In addition, recent data coming from the genomic analyses of patients-derived organoids isolated from colorectal cancer specimens suggest the existence of a core set of enhancers that are regulated by YAP/TAZ and are conserved in tumors of different sources but not in normal tissues or in cancer cell lines [98]. This tumor-specific enhancerome might constitute a pan-cancer genetic blueprint that implies a conserved YAP/TAZ function in determining cancer cells phenotypes [98].

## 5. Epigenetic Regulation

The evidence collected so far highlights a prominent role of epigenetic processes in the regulation of transcription by YAP/TAZ, both in Drosophila and in mammalian cells [38,99]. This regulation is mainly based on the recruitment of chromatin remodeling factors, which control the accessibility of regulatory regions (promoters and enhancers), and that of chromatin-modifying enzymes, which, depending on the context, may favor or prevent the recruitment of basal TFs and RNA polymerases. Both processes are thought to foster enhancer–promoter contacts and activity. The following is an overview of the epigenetic regulators known to contribute to YAP/TAZ-dependent transcriptional control.

*The SWI/SNF complex*. Genome-wide studies investigating chromatin structures in different cellular contexts report alterations in chromatin accessibility upon YAP/TAZ overexpression or depletion. Independent reports suggest that the SWI/SNF complex (switch/sucrose non-fermentable complex) might be a conserved chromatin remodeling partner of YAP/TAZ [100]. In Drosophila, Co-IP experiments coupled with mass spectrometry analysis revealed the physical interaction between Yki and the Brahma-associated protein (BAP), a component of the Drosophila SWI/SNF complex [101]. Depletion of Brahma impairs the regeneration of the midgut intestinal stem cell niche, thus suggesting that Yki-dependent control of intestinal stem cells proliferation and differentiation requires SWI/SNF [101]. Notably, ChIP data show the co-binding of Brahma, Scalloped (the Drosophila TEAD orthologue), and Yki at the promoter of target genes relevant for Yki-dependent proliferation [102]. In mammals, there is conflicting evidence concerning the role of the SWI/SNF complex in controlling YAP/TAZ-dependent transcription, possibly highlighting different levels of regulation. Proteomic studies in breast epithelial cells report that the WW domain of TAZ binds the PPXY motif of BRM, the catalytic ATPase subunit of the SWI/SNF complex, as well as other core components (BAF155, BAF170, and SNF5) [103]. These interactions are required to repress the luminal commitment of breast epithelial cells and to preserve their basal phenotype during lineage switching [103]. Overall, this would argue in favor of the association of YAP/TAZ with SWI/SNF in order to regulate chromatin accessibility. On the other hand, there is also strong evidence that the SWI/SNF complex acts as a sensor for nuclear mechanical stress and, by doing so, it is also able to modulate YAP/TAZ activity in a chromatin-independent manner [104]. Indeed, upon low mechanical tension, SWI/SNF interact with YAP/TAZ through the ARID1A subunit, preventing their association to TEADs. Conversely, upon high mechanical tension, the ARID1A–SWI/SNF complex is sequestered by the nuclear F-actin and actin-related proteins (Arps), thus allowing YAP/TAZ binding to TEADs [104]. Thus, YAP/TAZ are inhibited by the SWI/SNF complex in mechanically challenged cells. The genetic relationship between YAP/TAZ and the SWI/SNF complex is also complicated by the fact that SWI/SNF may also indirectly modulate YAP/TAZ function. For instance, in head and neck squamous cell carcinoma (HNSCC), the gene ACTL6A encoding for the human BAF–SWI/SNF subunit Brahma-associated factor 53a is recurrently amplified together with the TF p63. Their increased activity drastically reduces chromatin accessibility upstream of the transcription start sites of KIBRA, a known apical regulator of the Hippo pathway, leading to YAP/TAZ activation [105]. Further studies with more detailed biochemical characterization of SWI/SNF components coupled to context-specific genome-wide chromatin mapping will be beneficial to clarify some of open issues.

*p300 and BRD4*. The acetylation of chromatin at several histone residues is generally associated with transcriptional activation; importantly, YAP/TAZ are able to recruit selected histone acetyltransferases (HATs), mainly at enhancers, in order to activate gene expression. Enhancers bound by YAP/TAZ are heavily acetylated on H3K27 [36,43], a chromatin mark mainly deposited by the p300 lysine acetyltransferase. Both the silencing and inhibition of YAP lead to the loss of chromatin-associated p300 and the reduction of H3K27 acetylation, thus suggesting a fundamental role of YAP in activating enhancers by favoring the recruitment of p300. On the other hand, p300 silencing mirrors the loss of function of YAP, thus indicating a non-redundant role of p300 in YAP-dependent enhancers [36]. The recruitment of p300 by YAP/TAZ may also be relevant, when they co-adjuvate transcription driven by other TFs, as for a subset of p73-regulated promoters in response to DNA damage: YAP silencing impairs p300 recruitment at p73-regulated genes, reduces their histone acetylation and represses p73-driven apoptosis [106]. p300 may also co-activate YAP/TAZ-dependent transcription independently from its chromatin-associated function. For instance, during the TGF-β1 stimulation of hepatic stellate cells, cytoplasmic p300 associates with TAZ and SMAD2/3, and thanks to its nuclear localization signals, favors the nuclear import of the complex. Once in the nucleus, this complex induces chromatin acetylation and expression of TGF-β1 target genes [107].

YAP/TAZ can enhance chromatin acetylation by recruiting BRD4 [37,108]. BRD4 is a chromatin reader containing two bromodomains, which are required to bind acetylated histones, and a histone acetyltransferase catalytic domain located in the C-terminal part of the protein [109]. This catalytic domain has broad specificity for several residues located on histones’ tail and also for H3K122, which is located in the globular domain of H3. This latter event loosens histones binding to DNA, causing their eviction from chromatin [37]. By recruiting BRD4, YAP/TAZ favor enhancer–promoter contacts and increase the acetylation of H3K122 at the transcription start site of the enhancer-associated gene. This chromatin mark is essential for the recruitment and activation of RNA Pol II. Overall, we can envision that YAP/TAZ activate enhancers by recruiting p300 and BRD4, thus bookmarking chromatin to favor enhancer–promoter remodeling and promoting the binding and the activity of RNA-polymerase and its associated factors.

*Chromatin methylation*. Post-translational modifications of histones may affect their charge, impairing the affinity of DNA–nucleosomes binding and, thus, the degree of compaction of the chromatin structure. The methylation of lysine 4 of histone H3 is associated with gene activity: typically, H3K4me1 is observed at enhancers and H3K4me1 is observed at gene bodies, while H3K4me3 is abundant at promoters [110]. H3K4 methylation can be regulated by several histone methyltransferases (HMTs) such as Set1 (COMPASS), Trithorax (Trx), and Trithorax-related (Trr) proteins, which belong to different HMT complexes [111,112,113,114]. In human cells as well as in Drosophila, NCOA6, a subunit of the Trithorax-related MLL2/3 HMT complex, is an important player in regulating Hippo-mediated transcriptional responses, suggesting an evolutionarily conserved mechanism of gene expression regulation [115]. Firstly, Co-IP experiments coupled with mass spectrometry analysis unveil the direct interaction between the WW domain of Yki with the PPXY motifs of NCOA6. In addition, Yki ChIP-seq peaks extensively overlaps with Trr and H3K4me3 peaks, reinforcing the hypothesis that the Trithorax-related MLL2/3 HMT complex is recruited on Yki bound regions, where it catalyzes H3K4 methylation. Consistently, the overexpression or depletion of Ncoa6 mirrors the corresponding modulations in genes expression induced by Yki, thus suggesting that once Ncoa6 is recruited on Yki bound sites, it unwraps chromatin by histones methylation and promotes Yki-dependent transcription [114,115]. Evidence suggests that similar mechanisms control the expression of YAP/YAZ target genes in higher eukaryotes [114].

*The NuRD repressor complex*. The repression of transcription by YAP/TAZ is mainly associated with the recruitment of the NuRD complex, which possesses both histone deacetylase (HDAC) and ATP-dependent chromatin-remodeling activity [116]. This complex restricts genome accessibility around the bound regulatory regions, both by compacting nucleosomes through chromodomain helicase DNA-binding proteins 3/4 (CHD3/4) or by removing acetyl groups from lysine residues through histone deacetylase 1/2 (HDAC1/2) [117,118]. In human embryonic stem cells (hESCs), YAP/TAZ/TEAD recruit the NuRD complex on switch-enhancers, a subset of distal elements co-regulated by SMAD2/3 and OCT4. Their repression is needed for the maintenance of the pluripotent state of hESCs [73]. Similarly, in somatic cell lines, the repressive function of YAP/TAZ has been linked to the recruitment of the NuRD complex at the target loci [116]. Mechanistically, the deacetylation of histones around YAP/TAZ/TEAD–NuRD-binding sites increases the nucleosome occupancy, leading to the repression of transcription. As a result, genes involved in senescence or apoptosis are drastically silenced. In particular, the repression of TNF-related apoptosis-inducing ligand (TRAIL) is linked to increased cell survival, while the repression of DNA-damage-inducible transcript 4 (DDIT4) leads to the activation of the mechanistic target of rapamycin complex 1 (mTORC1), thus suggesting that YAP/TAZ-mediated gene repression may foster cell proliferation and survival [116].

## 6. Transcriptional Condensates and Phase Separation

YAP/TAZ can phase-separate to control transcription, possibly by forming transcription factories whereby distal enhancers, promoters, and transcriptional coactivators are co-opted by YAP/TAZ in order to activate gene transcription. YAP is shown to phase-separate in response to hyper-osmotic stress. YAP nuclear condensates are also identified in vivo in the kidney medulla as a result of the high osmolarity of this area. YAP condensates, which are driven by its intrinsically disordered transactivation domain, first form away from transcriptional complexes and then subsequently colocalize with nascent RNA once transcription is activated. These condensates are proximal but do not completely overlap with RNA Pol II signals, possibly hinting at their prominent role in shaping the spatial organization of enhancers, promoters, and their associated coactivators [119]. Similarly, TAZ phase-separates in the nucleus, when cells are stimulated with either serum, LPA, or EGF, or are grown on stiff substrates (i.e., high-cytoskeletal tension), all conditions known to activate TAZ. Coherently, the analysis of invasive breast cancer tissues revealed the presence of nuclear foci, suggesting that liquid–liquid phase separation may be particularly prominent when TAZ is strongly activated. The propensity of TAZ to phase-separate depends on the coiled-coil domain and, in part, on the integrity of the WW domain. These condensates colocalize with BRD4, MED1, CDK9, RNA Pol II, and H3K4me3, thus indicating the formation of fully active transcriptional foci [120]. Given that both reports acknowledge that YAP/TAZ-dependent transcription is active also in the absence of nuclear phase-separation, future work will be needed to clarify circumstances where nuclear condensates are needed and what are the gene programs, coactivators, and distal regulatory elements that are co-opted within these bonafide transcriptional hubs.

## 7. Hyper-Transcription and Transcriptional Addiction

Hyper-transcription is a state of a strong and global increase in RNA synthesis often associated with potent cell proliferation [121,122,123]. Hyper-transcription has been described during embryonic development, where it supports the biosynthetic demands of rapidly growing stem and progenitor cells [124] and also in cancer cells [125,126,127]. YAP/TAZ have been shown to drive hyper-transcription in the developing brain. Here, the loss of Lats1/2 in neural progenitor cells leads to the activation of YAP/TAZ, which in turn induces strong proliferation of stem and progenitor cells by controlling the transcription of genes involved in cell proliferation and cell growth [124]. In tumors, enhanced transcription, often dubbed as transcriptional amplification, has been linked to the engagement of oncogenic TFs such as MYC [126] and the broad epigenetic remodeling of the enhancer landscape [128]. This state, which is integral to the survival and fitness of cancer cells, leads to transcriptional addiction in tumors. In cancer cells, YAP/TAZ may control hyper-transcription by different means. On the one hand, YAP/TAZ are required for the recruitment of BRD4 on a broad range of enhancers and promoters of genes supporting cells proliferation and metabolism [37]. On the other hand, YAP/TAZ may support hyper-transcription by co-adjuvating the activity of growth controlling oncogenic TFs, such as E2Fs and MYC [37,56,126,129]. Indeed, genomic studies indicate that MYC could recruit YAP on a large fraction of its target genes, thus boosting MYC-dependent transcriptional output (and MYC oncogenic function) [45]. Importantly, the hyper-transcription and transcriptional addiction of cancer cells can be leveraged to design-targeted therapies. The basic transcriptional machinery engaged by YAP/TAZ may be selectively targeted, for instance, by using BRD4 inhibitors [37,130]. Similarly, considering that CDK7 promotes YAP stabilization (and activity) by directly phosphorylating and preventing their degradation, preclinically available CDK7 inhibitors could be considered to blunt YAP/TAZ-dependent transcription in cancer cells [41]. In addition, YAP/TAZ hyper-transcription has also been associated with replicative stress in developing neurons of adult hepatocytes, possibly due to the interference of transcription and DNA replication [131,132], thus potentially suggesting an intrinsic liability of YAP/YAZ-addicted tumors to replicative stress.

## 8. Transcriptional Program Controlled by YAP/TAZ in Cancer

**Figure 2:**
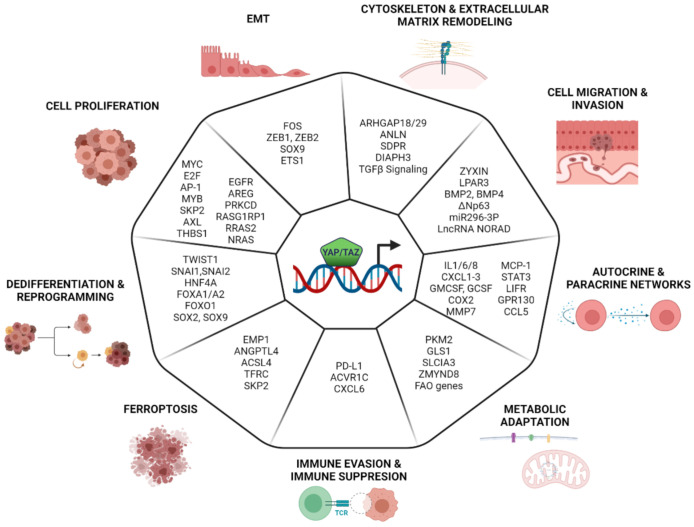
Cancer cell programs controlled by YAP and TAZ. This figure summarizes the biological processes controlled by YAP and TAZ in cancer cells. For each program, key downstream genes/pathways are reported according to their documented role as effectors of the indicated biological program. For the sake of simplicity, genes are all represented as regulated by YAP/YAZ, but it is implied that in many cases, the expression of these genes depends on the integration with other transcription factors, as detailed in the text. Created with BioRender.com. (https://help.biorender.com/en/articles/3619405-how-do-i-cite-biorender, accessed on 23 July 2021).

The dysregulation of YAP/TAZ has been reported in a wide variety of human cancers [133,134,135,136]. The majority of these reports accounts for a pro-tumorigenic/oncogenic function of YAP/TAZ, but it should be noted that there is also evidence for context-dependent tumor-suppressive activities [82,137,138]. The oncogenic activity of YAP/TAZ has been linked to enhanced cell survival, proliferation, invasiveness and metastasis, chemo-resistance, and promotion of stemness. The activation of YAP/TAZ is also associated with the remodeling of the tumor microenvironment and immune escape. Here, we report a summary of the emerging evidence linking the acquisition of these cancer hallmarks to the control of selected YAP/TAZ-dependent transcriptional programs (Figure 2).

### 8.1. Cell Proliferation

YAP/TAZ control cell proliferation by coordinating the expression of cell cycle genes, which constitute a core gene signature that almost invariably is activated by YAP/TAZ in multiple contexts. As previously discussed, this core cell cycle program is integrated by the activity of other TFs, such as MYC, E2F, AP-1, and MYB, both in normal and transformed cells [43,45,56,59,139]. YAP/TAZ also control the expression of these co-operating TFs [43,59,140,141]. Although it is reasonable to assume that proliferation is driven by the expression of a set of genes, in some cases, reports highlight the identification of downstream targets which are required to sustain YAP/TAZ-driven proliferation. For instance, YAP-driven proliferation in breast cancer cells depends on SKP2 expression: upon YAP inactivation, the downregulation of Skp2 and the consequential accumulation of p21 and p27 (which are degraded in a Skp2-dependent way) induce a cell cycle exit. Both Skp2 expression or p21/p27 depletion rescues proliferation in YAP-depleted cells [142]. Other YAP/TAZ transcriptional targets involved in the control of cellular proliferation are signal transduction genes. The epidermal growth factor receptor (EGFR) ligand amphiregulin (AREG) is shown to be required for YAP-induced proliferation in breast cancer cells [143], thus suggesting that EGFR signaling is an important YAP effector, regulating both physiological and malignant cell proliferation.

The AXL receptor tyrosine kinase is described as a key mediator of YAP-dependent oncogenic activities in human hepatocellular carcinoma (HCC). The ectopic expression of YAP increases expression levels of AXL protein and mRNA in the immortalized hepatocytes cell line MIHA. The knockdown of either YAP or AXL in primary HCC cell lines hampers tumor growth and metastasis [144]. Similarly, YAP overexpression stimulates spontaneous metastasis in experimental melanomas, while the silencing of YAP targets as AXL, THBS1 (Thrombospondin 1), and CYR61 represses metastasis. In this tumor type, while YAP activity is not associated with any of the recurrent genomic alterations, its transcriptional signature is strongly associated with the metastatic potential of melanomas cell lines and is linked to metastasis in patients datasets [145].

YAP/TAZ activity can also lead to the upregulation of the RAS/MAPK pathway: for example, in thyroid cancer whereby, due to NF2 inactivation, YAP leads to the increased expression levels of the three RAS genes and the activation of the RAS/MAPK pathway [146]. Similarly, in uveal melanoma cells, YAP induces the expression of Prkcd, Rras2, Nras, and RasGRP1, which is implicated in tumor initiation and progression [147], perhaps hinting at a general regulation of the RAS/MAPK signaling by YAP/TAZ.

### 8.2. EMT

Early studies based on the ectopic expression of either YAP or TAZ in human mammary epithelial cells showed the emergence of EMT phenotypes, including loss of cell–cell contact and cell scattering [148,149]. This is associated with the alteration of the expression of the classical EMT markers, such as E-cadherin and vimentin, and the transcriptional mediators Twist, Snail, and Slug. YAP/TAZ-driven EMT requires binding to TEADs, since the loss of function YAP/TAZ mutants or TEADs silencing impaires EMT, cell migration, and metastatic growth [27,28,150]. The regulation of EMT accounts, at least in part, for the ability of YAP to rescue oncogene addiction in Ras-dependent tumors, thus highlighting the relevance of the YAP/TAZ-dependent control of this process in cancer cells. Indeed, genetic experiments demonstrated that the ectopic expression of YAP is sufficient to rescue the loss of RAS in colorectal cancer cell lines. This is dependent on the ability of YAP/TEAD to engage the transcription of EMT and proliferative genes, in part co-regulated by FOS, a TF belonging to the AP-1 family. This is essential to overcome tumor attrition following RAS inactivation in lung tumors [151].

YAP/TAZ can control EMT at different levels, both by regulating the expression of EMT genes and master regulators and by establishing TFs regulatory networks. This dual control is exemplified by ZEB1, a transcriptional repressor essential for EMT. In skin squamous cell carcinomas, the hybrid-mesenchymal state, which is associated with aggressive metastatic growth and stemness, is due to the high expression of ZEB1. In these tumors, the loss of the protocadherin FAT1 leads to the activation of the CAMK2–CD44–SRC pathway that promotes YAP nuclear translocation, which in turn controls ZEB1 expression [152]. The detection of shared gene sets, co-regulated by ZEB1/YAP in breast cancers, suggests the existence of a coregulatory network that supports malignant cancer progression and therapy resistance. The direct interaction of ZEB1 with YAP (but not with TAZ) switches its function to an activator of a common ZEB1/YAP target gene set, known to stimulate cancer aggressiveness [46]. In particular, in the claudin-low subtype of aggressive breast cancer, the ZEB1/YAP/TEAD complex is shown to regulate the expression of canonical YAP/AP-1 targets and to activate genes programs linked to cell migration, cytoskeletal reorganization, and focal adhesion [66].

YAP/YAZ may also link the metabolic reprogramming of cancer cells to their acquisition of mesenchymal and migratory properties. A study on neural crest cells has recently shown that the Warburg effect could trigger EMT by promoting YAP1-TEAD1 nuclear interaction, which in turn regulates the activation of the expression of SOX9, ETS1, and ZEB2, key TFs driving neural crest delamination and migration. This is due to the YAP-dependent activation of enhancers [88]. This mechanism may link the Warburg effect to the acquisition of metastatic properties in aggressive/advanced tumors. A similar layered control may also link YAP/TAZ to the regulation of other EMT-TFs, such as SLUG and SNAIL.

### 8.3. Cytoskeleton and ECM Remodeling

While mechano-transduction is one of the main upstream pathways controlling YAP/TAZ activity, these two cofactors themselves control the expression of genes altering actin and cytoskeletal dynamics or remodeling the ECM [37,135,153]. This establishes a positive feedback regulation that potentially supports the continuous induction of YAP/TAZ in cancer cells. The remodeling of the cytoskeleton may also be required to confer peculiar properties to cancer cells. For instance, flexibility in the cytoskeleton may regulate YAP/TAZ to facilitate cancer cell migration and their systemic dissemination [19,154,155]. In gastric cancer, YAP promotes the expression of ARHGAP29, a RhoGAP that suppresses the RhoA-LIMK-cofilin pathway. This induces F-actin depolymerization, which reduces cytoskeletal rigidity and promotes metastasis [156]. Similarly, YAP is able to regulate tissue tension and fibronectin assembly by increasing ARHGAP18 transcription in human retina-pigmented epithelial cell lines [157].

The regulation of the cytoskeleton is also relevant within the non-tumor component of the tumor microenvironment: the activation of YAP in cancer-associated fibroblasts (CAFs) is required for promoting matrix stiffening, cancer cell invasion, and angiogenesis. In CAFs, YAP, apart from the well-known target genes AMOTL2, ANKRD1, and CTGF, regulates the expression of several cytoskeletal regulators, including ANLN, SDPR, and DIAPH3 [158]. The regulation of cytoskeletal and matrix remodeling programs may require the integration of YAP/TAZ activity with the Myocardin-related TFs (MRTFs), since synergistic gene activation by YAP/TAZ and MRTFs was reported for a subset of their respective target genes, in both fibroblast and CAFs. These are enriched in genes controlling cytoskeletal dynamics, thus establishing positive feedback and cross-regulation of both complexes (i.e., on YAP/TAZ or MRTF selective targets), which rendered the two pathways interdependent: YAP/TAZ potentiates MRTFs activity via TGF-β signaling, while MRTFs activates YAP/TAZ, via the upregulation of the Septin regulator Cdc42ep3 [60]. In addition, MRTFs, by binding YAP/TEAD, promote their transcriptional activity. MRTF–YAP binding is also triggered by acute actin cytoskeletal damage in a LATS-independent way. This suggests a Hippo-independent mechanism for the activation of YAP in both metastatic cancer cells and in CAFs [61].

### 8.4. Cell Migration and Invasion

The initial evidence of the involvement of YAP/TAZ in determining the migratory and invasive phenotype of cancer cells comes from Lamar et al., where multiplexed in-vivo assays (Luminex-based) show the pro-metastatic function of YAP in breast and melanoma tumors. This is dependent on the interaction with TEAD, and the increased metastatic activity on these cells correlates with increased YAP transcriptional activity [159]. It is important to note that both the migratory and invasive properties conveyed by YAP/YAZ are almost invariably associated with EMT and cytoskeletal remodeling; thus, these programs are not easily teased apart. This notwithstanding, there are some studies that have provided clues concerning the YAP/YAZ target genes required for migration and invasion. For instance, the expression analysis of TGF-β induces genes during EMT coupled to ChIP-seq analyses leads to the identification of zyxin as a target of TAZ/TEAD2, which is relevant for cell migration. Zyxin is a component of focal adhesions and also an actin cytoskeleton-remodeling protein. It localizes to sites of focal adhesions and stress fibers in response to mechanical cues to facilitate actin polymerization and the generation of traction force. The silencing of zyxin blocks migration and invasion in in vitro assays [160]. Similarly, YAP expression boosts migration and the invasive phenotype of metastatic prostate-ductal adenocarcinomas (PDAC). The overexpression of activated mutant YAP reveals that its metastatic activity depends, at least in part, on the upregulation of the LPA receptor 3 (LPAR3) [161]. This suggests a positive feedback loop whereby YAP sustains the metastatic growth by triggering the expression of LPAR, a class of G-protein-coupled receptors that are known to regulate YAP/TAZ [161].

Bone morphogenic protein 4 (BMP4) is identified as a direct target of TAZ in MCF10A. BMP4 upregulation associated with increased BMP4-SMAD1/5 signaling and BMP4 stimulation is sufficient to induce cell migration. BMP4 silencing partially inhibits TAZ-induced migration, suggesting that BMP4 signaling is one of the pathways engaged by TAZ to confer migratory properties to cancer cells [162].

Whole-genome profiling analysis leads to the identification and validation of target genes repressed by TAZ that may play a role in regulating cell migration [163]. In breast and lung epithelial cells, the p63 isoform ΔNp63 is significantly downregulated by TAZ overexpression. The suppression of ΔNp63 by TAZ/TEAD reduces cell–cell adhesion and increases cell migration [163].

Besides, YAP can affect cell migration and tumor metastatic dissemination by controlling the expression of miRNAs and long noncoding RNAs (lncRNAs). Recently, it was published that the YAP/TEAD/NuRD complex can repress the LncRNA NORAD. Mechanistic studies suggest that NORAD sponges several proteins involved in cellular migration; among these, S100P, a calcium-binding protein, is a prominent target required for the NORAD-mediated suppression of metastasis. NORAD expression is negatively correlated with prognosis and metastatic progression in breast and lung cancer, possibly suggesting a general role of this lncRNA in cancer [164]. In glioblastomas, YAP confers invasiveness by upregulating miR296-3p as well as other canonical YAP targets (neuronal growth regulator 1 (NEGR1), matrix metalloproteinase 1 (MMP1), endothelin 1 (EDN1), CYR61, and CTGF). Mechanistically, miR296-3p suppresses the STAT5A/SOCS2 pathway, thus favoring the activation of the TF STAT3, which in turn enhances the invasiveness of glioblastoma cells. The silencing of either CYR61 or miR296-3p represses the increased invasiveness induced by either NF2 loss or the activation of YAP. The retrospective analysis of clinical data indicates a poor prognosis for patients with high expression levels of both CYR16 and miR296-3p [165].

### 8.5. Metabolic Adaptation

In order to survive in hostile microenvironments characterized by poor nutrient availability, cancer cells must reprogram their cellular metabolism to fulfill their needs for rapid growth. These crucial adaptive responses are at least partially regulated by YAP/TAZ.

The investigation of the connection between metabolic reprogramming and metastatic growth in isogenic colon cancer cell lines reveals a positive regulatory loop linking glucose metabolism and metastatic growth. In these cells, YAP upregulates the glucose transporter GLU3, thus leading to increased glucose uptake. This activates the glycolytic enzyme pyruvate kinase 2 (PKM2), which is associated with YAP/TEAD, and increased their transcriptional activity. The YAP/TEAD/PKM2 complex drives the expression of genes linked to glycolysis, nucleotide synthesis, and glutathione-dependent detoxification pathways. The silencing of either YAP or GLUT3 leads to the reduction of metastatic growth in vivo, underscoring how the metastatic phenotype may depend on glucose-driven metabolic reprogramming of cancer cells. In the light of previous evidence showing that glycolysis can activate YAP through AMPK and PFK1 [166,167], pleiotropic YAP activation by glucose metabolism may represent a general theme in metastasis [168].

Bertero and collaborators described a metabolic switch triggered by the stiffening of the tumor microenvironment, which is essential for cancer progression and metastasis. YAP/TAZ regulate the expression of GLS1 (glutaminase) and the aspartate/glutamate transporter SLC1A3, both in tumor cells and in cancer-associated fibroblasts. This boost in glutaminolysis is needed for the TCA cycle-dependent regulation of amino acid biosynthesis and other biosynthetic pathways [169]. Lipid metabolism is also regulated by YAP/TAZ. In lymph nodes–metastatic tumors, YAP activity is required to support the expression of several genes implicated in fatty acid oxidation (FAO). The accumulation of bile acids is able to activate YAP, mainly via the nuclear vitamin D receptor, and leads to increased FAO. The knockdown of YAP significantly reduces FAO in the metastasis-adapted cells and suppresses their growth. The regulation of FAO genes by YAP seems specific for metastatic tumors infiltrating the lymph nodes, since it is not observed in primary tumors or in lung metastasis. This is supported by clinical data showing increased levels of nuclear YAP (i.e., activated) in metastatic cells found in lymph nodes [170]. These results suggest that YAP activation is a key molecular event that mediates FAO activation in metastatic lymph nodes. In Lgr5^+^ intestinal stem cells and in colorectal cancer cells, YAP can regulate cholesterol metabolism by controlling the expression of ZMYND8, a TF of the zinc finger-MYND family. In these cells, ZMYND8 associates to SERBP2 to drive enhancer–promoter interaction and thus upregulates genes of the mevalonate pathway, the main metabolic pathway controlling sterol synthesis. The loss of ZMYND leads to the demise of intestinal stem cells and cancer cells and is associated with loss of intestinal regeneration and delayed tumorigenesis [171]. This activation may serve the dual purpose of providing the metabolic needs of the cells and at the same time support the “cholesterol-driven” activation of YAP/TAZ [172]. Thus, YAP controls glucose metabolism, anaplerotic pathways regulating the TCA cycle, and lipid metabolism. It is interesting to note how metabolic control by YAP may serve the dual purpose of supporting the metabolic needs of the cell and, at the same time, may provide the feedback regulation of YAP/YAZ. This is the case for sterol biosynthesis, where increased sterol production activates YAP/TAZ [172] or, as in the case of glycolytic flux, where glucose metabolic enzymes may increase the activity of YAP/YAZ [168].

### 8.6. Autocrine and Paracrine Signaling

A relevant part of the transcriptional programs controlled by YAP/YAZ in cancer cells concerns the establishment of paracrine and autocrine signaling. In particular, expression profiling and loss of function studies in MDA-MB-231 cells have allowed the identification of a set of cytokines (IL1, IL6, IL8, and CXCL1-3) and hematopoietic growth factors (GMCSF and GCSF), of which the expression levels are dependent on YAP/TAZ. This is associated with the activation of STAT3 and NFKB signaling. The supplementation of these cytokines promotes invasiveness in in vitro assays while blocking CXCR2 signaling (CXCR2 is the receptor for CXCL1-3 and IL8), loosens the endothelial monolayer and promotes invasion and extravasation [173]. This pairs with observations that restoring LIFR expression in invasive breast cancer cell lines inhibits both YAP activity and metastasis [174]. Modeling KRAS-dependent pancreatic ductal adenocarcinomas leads to the identification of a secretory program including COX2, MMP7, IL-6, and IL-1α, which is supported by YAP, along with the canonical YAP/TAZ targets CTGF and CYR61. Considering the role of these genes in supporting tumor-associated stromal responses, this may indicate a role for YAP in remodeling the tumor-associated stroma. Indeed, the deletion of YAP dampens both the recruitment and the activation of CAFs, and the production of collagen, thus leading to the reduced proliferation of cancer cells. Of note, conditioned media from these tumors restore proliferation in YAP-deficient tumors [175]. YAP/TAZ may also regulate the intracellular signaling of inflammatory pathways. In murine models of pancreatic cancer progression, YAP/TAZ induce the expression of genes linked to IL6/JAK/STAT signaling. In particular, STAT3, LIFR, and GPR130 (IL6 co-receptor) are direct YAP/TAZ targets upregulated by oncogenic RAS. YAP/TAZ deletion blunts pancreatic inflammation and adeno-ductal metaplasia driven by KRAS [176]. Considering that GP130 also activates YAP independently of STAT3 [177], this may represent a positive feedback loop amplifying YAP activity. These data suggest that YAP/TAZ act upstream of the JAK-STAT3 signaling pathway in RAS-induced acinar-to-ductal metaplasia and that, even in the absence of systemic inflammation, oncogenic RAS can lead to YAP/TAZ and JAK–STAT3 activation [176]. The crosstalk of YAP/TAZ signaling and JAK/STAT is also highlighted by their coordinated upregulation by ABL kinases in metastatic breast cancer, which is needed and sufficient to promote bone metastasis in experimental models [178]. YAP/TAZ-driven paracrine signaling is also implicated in the interaction between cancer cells and tumor-associated macrophages (TAMs). The crosstalk of breast cancer cells and TAMs leads to the stabilization of YAP due to the activation of OTUD-5, a deubiquitinating enzyme that prevents the ubiquitin-dependent degradation of YAP. Gain of function and loss of function experiments suggest that YAP is required for M2-polarization and that the overexpression of YAP in M2-macrophages potentiates their pro-invasive phenotype in breast cancer cells. Among the cytokines induced by YAP, MCP-1 and its cognate receptor CCR2 (expressed by tumor cells) are critical to enhance migratory and invasive properties of triple-negative breast cancer cells [179]. Similarly, in clear cell renal cell carcinoma (ccRCC), a positive regulatory loop, supported by YAP and STAT3, facilitates the interaction between ccRCC tumor cells and TAM. The downregulation of SOX17 in renal cells promotes YAP activity and the secretion of the cytokine CCL5. CCL5 exerts a dual role: (i) paracrine stimulation of macrophages and induction of their polarization towards TAM phenotype; and (ii) autocrine stimulation, whereby CCL5/CCR5/STAT3 signaling supports YAP activation and suppresses SOX17 expression [180]. This crosstalk between cancer cells and TAMs is required for tumor growth, drug resistance, and metastatic dissemination.

### 8.7. Immune Evasion and Immune Suppression

Within the tumor microenvironment, cancer cells interact with immune cells often to evade or suppress the immune system. There is emerging evidence that YAP/TAZ may trigger immunosuppression by diverse mechanisms. In tumor cells, YAP/TAZ can decrease the expression of programmed death-ligand 1 (PD-L1), a surface protein that activates the immune checkpoint, thus contributing to the repression of T-cell function [181,182,183]. Furthermore, YAP is highly expressed in regulatory T-cells (T-reg) and can boost their immunosuppressive activity by controlling the expression of Acvr1c (activin), which serves as an amplifier of TGF-β signaling. Consequently, YAP-deficient T-reg displays the reduced expression of TGFβ-dependent anti-inflammatory cytokines and shows impaired immune suppressive function. Thus, the loss or inhibition of YAP in T-cells augments the anti-tumor immune response, either as a monotherapy or in combination with tumor vaccines or anti-PD-1 treatment [184]. YAP/TAZ may also regulate the recruitment of myeloid-derived suppressor cells (MDSCs) within tumors. MDSCs are immature myeloid cells that support tumor growth by maintaining a state of immunologic anergy and tolerance. Within tumors, activated MDSCs provide a source of secreted chemokines, cytokines, and enzymes, which suppress local T-cell activation and viability through the deprivation of nutrients (L-arginine and L-cysteine) and interfere with T-cell receptor functions via reactive oxygen species and reactive nitrogen species. In a mouse model of prostate tumors, YAP regulates the expression of the Cxcl5 chemokine (CXCL6 in humans), which is required to activate the recruitment of MDSCs via the CXCR2 membrane receptor. The inhibition of CXCR2 expression in MDSCs or YAP silencing in tumor cells inhibits tumor growth, suggesting that YAP-mediated recruitment of MDSCs is required for tumor progression. The observation that the MDSC gene signature is enriched in human prostate cancers with high YAP activity suggests a conserved etiological role in human tumors [185].

### 8.8. Ferroptosis

Ferroptosis is a novel type of regulated cell death, which is iron-dependent and characterized by the accumulation of peroxidated lipids [186,187]. The exhaustion of intracellular glutathione or excessive iron originated from aberrant iron metabolism specifically triggers lipid peroxidation and ferroptosis due to accumulation of oxidative radicals [186,188,189]. This is balanced by glutathione peroxidase 4 (GPX4), which protects cells from ferroptosis by neutralizing the accumulation of lipid peroxidation that results from the oxidative stress generated by the NADPH oxidases (NOXs) [186,190]. Thus, the inhibition of GPX4, either directly or indirectly, by depriving its cofactor glutathione or building blocks of glutathione (such as cysteine), is one of the main triggers for ferroptosis. Cancer cells may display higher sensitivity to ferroptosis due to their higher dependency on iron, a dependency that could be leveraged for therapeutic purposes [188,191,192].

Recent studies have revealed that YAP/TAZ can promote ferroptosis in cancer cells. Yang et al. showed that in renal cell carcinomas, ferroptosis susceptibility is regulated by cell density and confluency. Mechanistically, low cell density increases TAZ activity and leads to the accumulation of the epithelial membrane protein 1 (EMP1), which, in turn, induces the expression of NADPH oxidase 4 (NOX4), a key regulator of lipid peroxidation for ferroptosis [193]. A similar mechanism is described in ovarian cancer cells, where the loss of cell–cell contacts leads to the activation of TAZ, which induces Angiopoietin-like 4 protein (ANGPTL4) that sensitizes ferroptosis by activating the NADPH oxidase 2 (NOX2) [194]. In epithelial cells, YAP/TEAD promote the upregulation of the ferroptosis modulators acyl-CoA synthetase long-chain family member 4 (ACSL4) and the transferrin receptor (TFRC) [192]. Other recent integrative analyses identified the E3 ubiquitin ligase SKP2 as a YAP–TEAD direct target gene that regulates ferroptosis in renal and ovarian cancer. SKP2 downregulation leads to the stabilization of the protein kinase TTK and TFRC, thus favoring the ferroptosis protection [195].

Overall, this suggests that ferroptosis may be a selective liability of YAP/TAZ-driven tumors that could be exploited for therapeutic purposes.

### 8.9. De-Differentiation and Reprogramming

De-differentiation is a process that confers plasticity to cells, thereby enhancing adaptive responses, in particular during development, tissue regeneration, and oncogenic transformation. This process, crucial for the function of progenitors and stem cells, is often hijacked during tumor evolution to sustain tumor progression towards more aggressive and metastatic states and to promote drug resistance [196,197,198]. The first evidence linking the activation of YAP/TAZ to de-differentiation comes from the gain of function studies in animal models, where tissue overgrowth is associated with the expansion of progenitor-like/stem cells [199,200,201,202]. Follow-up studies confirm the role of YAP/YAZ in regulating progenitors and stem cells in mammals, particularly in the intestinal epithelium [203,204]. In the adult intestine, YAP expression is restricted to the stem cell niche at the bottom of the crypt, and ectopic expression of an activated YAP can lead to a loss of differentiated cell types in the small intestine and results in a rapidly and reversibly expansion of multipotent undifferentiated intestinal progenitor cells [199]. The loss of both YAP and TAZ does not affect the homeostasis of the intestinal epithelium but strongly limits its regeneration [205]. This is because, during regeneration, Yap transiently reprograms Lgr5+ intestinal stem cells by suppressing Wnt signaling and excessive Paneth cell differentiation and, at the same time, induces a regenerative response driven by the activation of the EGF pathway [205]. In addition, YAP contributes to regeneration by expanding a second reservoir of highly quiescent stem cells (i.e., revival stem cells), which can replenish the Lgr5+ intestinal stem cells and fuel transit-amplifying cells production [206]. Similarly, the activation of YAP/TAZ in the adult liver promotes hepatocytes de-differentiation and the emergence of bipotent progenitor’s cells, which is supported by high Notch signaling and shows remarkable engraftment and regenerative potential [202]. The reprogramming of adult somatic cells to undifferentiated multipotent progenitors may be a general property of YAP/TAZ, as suggested by their broad capability to convert differentiated cells into tissue-specific stem/progenitor cells [207]. YAP/TAZ activity is also required to sustain self-renewal and tumor-initiation capacities of cancer stem cells (CSCs) with implications on tumor initiation, cell plasticity, drug resistance, and metastasis [13,208,209,210]. As a result, YAP/TAZ act as central regulators of genes responsible for cellular de-differentiation and regeneration in several tumor types. In breast cancer, TAZ activity is particularly high in poorly differentiated and aggressive tumors, where it controls the expression of a signature of stem cells genes. The activation of TAZ, due to EMT induction or the loss of the epithelial polarity protein Scribble, endows non-CSCs with self-renewal capacity [13]. The hyperactivation of YAP is considered a major oncogenic event in embryonal rhabdomyosarcoma [211]. In this tumor, YAP–TEAD1 block the myogenic differentiation gene program led by the TFs MYOD1 and MEF. This is due to both the direct repression of MYOD1/MEF target genes, which are bound by TEAD1 and by the YAP-dependent upregulation of TWIST1, SNAI1, and SNAI2, which are known antagonists of MYOD1/MEF. Thus, YAP is able to upregulate oncogenic and pro-proliferative genes and maintain cancer cells undifferentiated [211]. Defective YAP signaling in the liver results in the development of HCC [140]. Changes in YAP activity may reprogram subsets of hepatocytes to different fates associated with the deregulation of HNF4A, α-catenin, and E2F transcriptional programs, thus preventing hepatocyte quiescence and differentiation. Transcriptomic analyses suggest that YAP represses the expression of HNF4A and FOXA1/FOXA2 targets, in both normal liver and HCC. Accordingly, the suppression of HNF4A activity sustains both cell proliferation and the block of differentiation in in vivo models [140]. YAP/TAZ are also shown to mediate differentiation, stemness, and cell plasticity in glioblastoma [78]. Single-cell RNA-seq and computational analyses showed that YAP/TAZ act as master regulators of the glioblastoma stem-like cells (GSCs) by controlling a regulatory network orchestrated by FOXO1. During glioblastoma initiation, YAP/TAZ activation represses the differentiation of normal neural cells and promotes the upregulation of a set of neural stem cell genes. Moreover, in vitro and in vivo losses of function experiments show that YAP/TAZ ablation downregulates neural stem cell markers and upregulates neural differentiation markers, suggesting that YAP/TAZ are required to prevent the differentiation of GSCs [78]. Tumor cell stemness may also be promoted by the concerted dysregulation of YAP/TAZ along with other TFs. YAP can co-operate with Oct4 to regulate self-renewal by modulating SOX2 expression in non-small lung cancer cells. The overexpression of Sox2 in YAP null cells rescues the loss of CSCs and tumor growth features, suggesting that Sox2 is the essential downstream effector of YAP. Interestingly, YAP could directly interact with the Oct4 TF to induce Sox2 expression [212]. Likewise, the genetic activation of YAP is associated with stemness in esophageal squamous cell carcinomas (ESCC). Mechanistically, YAP induces the expression of SOX9 via TEAD1-mediated binding to confer CSC-like features to ESCC cells [213]. In summary, these and many other reports suggest that YAP/TAZ exert a broad control of stemness and de-differentiation in a wide variety of tumors.

## 9. Conclusions and Perspectives

We have certainly gained considerable knowledge of how YAP/TAZ control transcription. Overall, it is generally accepted that YAP/TAZ play a pivotal role in controlling distal enhancers and in linking enhancer activation to their associated genes. This implies a crucial role for mechanisms in controlling three-dimensional (3D) chromatin organization and looping, which must be engaged or at least negotiated by YAP/TAZ in order to shape and integrate transcriptional responses. Understanding these processes is undoubtedly one of the key challenges that lie ahead. It is also emerging that YAP/TAZ are predominantly controlling transcription by epigenetic mechanisms and by establishing an extensive network of functional and physical interaction with several TFs which orchestrate context-dependent transcriptional responses. Further biochemical studies and genome-wide chromatin mapping will be essential to extend and possibly complete the entire catalog of TFs, cofactors, and chromatin modifiers that participate in YAP/TAZ-dependent transcriptional regulation. Considering that YAP/TAZ sit at the crossroads of signaling pathways controlling development, regeneration, and cancer, a full description of their context-dependent activity as transcriptional cofactors will offer a unique opportunity for designing molecular therapies to modulate their function in the relevant clinical settings.
